# Prediction model for compressive strength of alkali-activated multi-source solid-waste grouting materials based on generative adversarial networks and machine learning

**DOI:** 10.1371/journal.pone.0357051

**Published:** 2026-09-08

**Authors:** Qiang Yue, Guangming Yu, Yong Yao, Zhiqi Yang, Yufeng Li, Liyang Bai, Guangwei Xiong

**Affiliations:** 1 China Construction Fifth Engineering Bureau Co., Ltd., Changsha, Hunan, China; 2 School of Civil Engineering, Qingdao University of Technology, Qingdao, Shandong, China; 3 Department of Resources and Mechanical Engineering, Lvliang University, Lvliang, Shanxi, China; Instituto Federal do Espírito Santo: Instituto Federal de Educacao Ciencia e Tecnologia do Espirito Santo, BRAZIL

## Abstract

Alkali-activated multi-source solid-waste grouting materials have attracted increasing attention in geotechnical and underground engineering due to their low carbon footprint, environmental friendliness, and potential for large-scale resource utilization. However, the complex composition and multiple influencing factors of these materials pose significant challenges for understanding and predicting their compressive strength, particularly under conditions characterized by small sample sizes, high-dimensional variables, and strong nonlinearity. To address these issues, an experimental database comprising 179 UCS records compiled from three independent experimental studies was established. Fifteen experimental records were reserved for external validation, while WGAN-GP was trained using the remaining 164 model-development samples, thereby keeping the external validation set completely isolated from model development. Multiple machine learning models were then developed to establish a UCS prediction framework. Furthermore, the Newton–Raphson-Based Optimizer (NRBO) was applied to optimize the hyperparameters of the XGBoost model, resulting in an NRBO-XGBoost prediction model. The SHAP method was introduced to interpret the prediction results and quantify the contribution of each input variable. The results demonstrate that WGAN-GP effectively preserves the statistical characteristics and inter-variable correlations of the original data, thereby improving model-training stability and generalization performance. Among all models considered, NRBO-XGBoost exhibited the best predictive performance, achieving an R^2^ of 0.964, an RMSE of 3.411 MPa, and an MAE of 2.474 MPa in the testing phase. SHAP analysis further revealed that cement content, water-to-binder ratio, curing age, alkali equivalent, and ground granulated blast-furnace slag content play dominant roles in governing compressive strength development. The integrated framework of data augmentation, optimized modeling, and interpretable analysis proposed in this study provides reliable data-driven decision support for mix design optimization and engineering application of alkali-activated multi-source solid-waste grouting materials, and offers a novel technical pathway for performance prediction of complex cementitious materials.

## 1. Introduction

Grouting materials are widely used in geotechnical and underground engineering and play a crucial role in tunnel construction, surrounding rock stabilization, and seepage control in subsea tunnels. The mechanical properties of grouting materials are directly related to the overall stability and service safety of engineering structures, among which the unconfined compressive strength (UCS) is regarded as one of the core indicators for evaluating the load-bearing capacity and structural integrity of grouting materials [1]. At present, the grouting materials commonly used in engineering practice are still dominated by Ordinary Portland Cement (OPC). However, its production process is characterized by high energy consumption and significant carbon emissions, which has become a major constraint on the low-carbon transition and sustainable development of the construction industry [2,3].

In recent years, with the advancement of the “dual-carbon” goals and the concept of the circular economy, alkali-activated materials (AACMs) based on industrial solid wastes have attracted increasing attention from researchers both domestically and internationally. Studies have shown that industrial by-products rich in aluminosilicate components, such as ground granulated blast furnace slag (GGBS), fly ash (FA), and steel slag (SS), can form structural systems with favorable cementitious properties under alkaline activation. This approach not only enables the large-scale valorization of industrial solid wastes but also significantly reduces carbon emissions and environmental burdens throughout the material life cycle [4–6].

However, the strength formation mechanism and performance evolution of alkali-activated multi-source solid-waste grouting materials are highly complex. Such material systems usually contain multiple solid-waste precursors simultaneously, whose chemical compositions, mineralogical phases, and reaction activities differ significantly, leading to complex synergistic reactions under alkaline activation conditions [7–9]. Previous studies have demonstrated that the compressive strength of these materials is not only closely related to the type and dosage of solid wastes but is also governed by the coupled effects of multiple factors, including alkali dosage, alkali activator modulus, water-to-binder ratio, curing age, and curing regime [10,11]. Although conventional experimental approaches can directly characterize the mechanical properties of materials, they suffer from high cost and long testing periods under multi-factor and high-dimensional parameter spaces, and they are limited in their ability to systematically capture the nonlinear relationships among variables [12]. Moreover, due to the difficulty in obtaining engineering experimental data, the datasets available for modeling and analysis in existing studies are generally small, resulting in empirical formulas or regression models with limited applicability and insufficient accuracy in predicting the compressive strength of different mix designs [13–15].

Against this background, developing models capable of accurately predicting the compressive strength of alkali-activated multi-source solid-waste grouting materials is of great significance for improving the efficiency of solid-waste resource utilization, optimizing mix proportion design, and promoting their engineering applications. A systematic investigation of the mechanical properties of such materials and their influencing factors not only facilitates the development of green grouting materials but also provides important theoretical support and technical pathways for achieving low-carbon and intelligent design in the field of geotechnical engineering.

With the rapid development of data science and artificial intelligence technologies, machine learning (ML) methods have gradually been introduced into civil engineering and material property prediction due to their advantages in nonlinear modeling and high-dimensional data processing. Models such as support vector regression (SVR), random forest (RF), extreme gradient boosting (XGBoost), light gradient boosting machine (LightGBM), and artificial neural networks (ANN) have been successfully applied to the prediction of compressive strength for concrete, geopolymers, and alkali-activated materials [14,16,17]. Recent studies have further demonstrated the effectiveness of optimized and interpretable ML models for strength prediction in alkali-activated and geopolymer materials. Fang et al. developed an optimized XGBoost model for predicting the compressive strength of alkali-activated concrete, while Ahadian et al. applied interpretable ML to fly ash-based geopolymer concrete. More closely related to the present study, Wang et al. combined optimized ML and SHAP analysis to predict the compressive strength of ternary industrial-waste-based geopolymer grouting materials [18–20].

Related studies indicate that ensemble learning models generally exhibit higher predictive accuracy and better generalization capability in complex material systems [21,22]. Moreover, gradient-boosting models combined with conformal prediction have recently been applied to uncertainty-aware prediction of chloride permeability in SCM-blended concrete and release behavior in glass-ionomer cement systems [23,24]. However, the performance of machine learning models is highly dependent on data quantity and quality, and they are prone to overfitting and instability under small-sample conditions. In addition, model hyperparameter settings have a significant impact on prediction performance, while traditional grid search or empirical tuning methods are often inefficient and struggle to obtain global optimal solutions [25].

To alleviate the limitations imposed by insufficient experimental data on machine learning modeling, data augmentation techniques have attracted increasing attention. Generative adversarial networks (GANs), first proposed by Goodfellow et al., are capable of generating high-quality synthetic samples by learning the distribution of original data, and have achieved remarkable success in image processing, signal analysis, and engineering data modeling [26,27]. In recent years, GANs have been introduced into materials science and structural engineering to expand limited experimental datasets, thereby improving the stability and generalization performance of predictive models. Among them, the Wasserstein GAN with gradient penalty (WGAN-GP) effectively addresses training instability and mode collapse issues commonly encountered in traditional GANs by incorporating the Wasserstein distance and gradient penalty mechanism, and has demonstrated superior performance in small-sample data augmentation [28–30]. Existing studies have shown that combining GAN-based data augmentation with machine learning models can significantly enhance the accuracy of compressive strength prediction for concrete and geopolymer materials [31,32]; however, systematic investigations of this approach for alkali-activated multi-source solid-waste grouting materials remain limited.

To address these issues, this study constructs a compressive strength database for alkali-activated multi-source solid-waste grouting materials based on experimental data. A Wasserstein generative adversarial network (WGAN-GP) is introduced to perform high-quality augmentation of the original small-sample dataset, and multiple machine learning models are integrated to establish a compressive strength prediction framework. Furthermore, the Newton-Raphson-based optimization algorithm (NRBO) is employed to optimize the hyperparameters of the XGBoost model, thereby further enhancing its predictive accuracy and generalization capability. Meanwhile, the SHapley Additive exPlanations (SHAP) interpretability method is adopted to systematically reveal the contribution and influence patterns of each input variable on the predicted compressive strength. The findings of this study provide reliable data-driven decision support for mix proportion optimization and engineering applications of alkali-activated multi-source solid-waste grouting materials, and offer a novel technical perspective for performance prediction of complex cementitious materials.

## 2. Methodology

### 2.1. Dataset

The dataset used in this study was compiled from three experimental investigations [33–35], and all data were collected under compliant and well-defined parameter conditions. The complete original experimental dataset is provided in S1 Data. The original dataset consisted of 179 experimental UCS records of alkali-activated multi-source solid-waste grouting materials. Each record contained eight input variables, namely cement content, steel slag (SS) content, ground granulated blast furnace slag (GGBS) content, fly ash (FA) content, alkali equivalent (AE), alkali activator modulus, water-to-binder ratio (W/B), and curing age, while the unconfined compressive strength (UCS) was adopted as the output variable. Before model development, 15 independent experimental samples were reserved as an external validation set and were not involved in any subsequent data augmentation or model training process. The remaining 164 samples were used as the model-development dataset for two comparative modeling schemes. In the original-data modeling scheme, the 164 samples were randomly divided into training and testing subsets at a ratio of 8:2, and machine learning models were trained and evaluated using only the original experimental data. In the augmented-data modeling scheme, the WGAN-GP model was trained using the 164 model-development samples, and 2000 synthetic samples were generated under predefined physical and parameter constraints. The augmented dataset, consisting of the 164 original samples and 2000 synthetic samples, was then used to train the machine learning models. The reserved 15 independent experimental samples were excluded from data generation, normalization parameter estimation, hyperparameter optimization, and model training, and were used only for external validation. This strategy enabled a comparative evaluation between the original-data-based and augmented-data-based models while preventing information leakage from the external validation set. Table 1 lists the variable names, units, and statistical characteristics, demonstrating the wide range of binder compositions and mixture proportions represented in the dataset.

**Table 1:** Statistical characteristics of the original experimental dataset.

Features	Units	Min	Max	Mean	Std	C.V.
cement	%	0	100	24.58	22.72	0.92
SS	%	0	60	17.21	15.32	0.89
GGBS	%	0	100	52.18	16.7	0.32
FA	%	0	40	6.03	12.2	2.02
Alkali equivalent	–	2	12	6.3	1.81	0.29
Alkali activator modulus	–	0.8	1.8	1.46	0.24	0.16
W/B	–	0.32	0.8	0.64	0.2	0.31
Age	days	1	28	13.28	11.07	0.83
UCS	MPa	2.2	86.2	27.53	18.32	0.66

Note: The dataset contains 179 experimental records. Fifteen records were reserved as an external validation set before model development.

**Fig 1:**
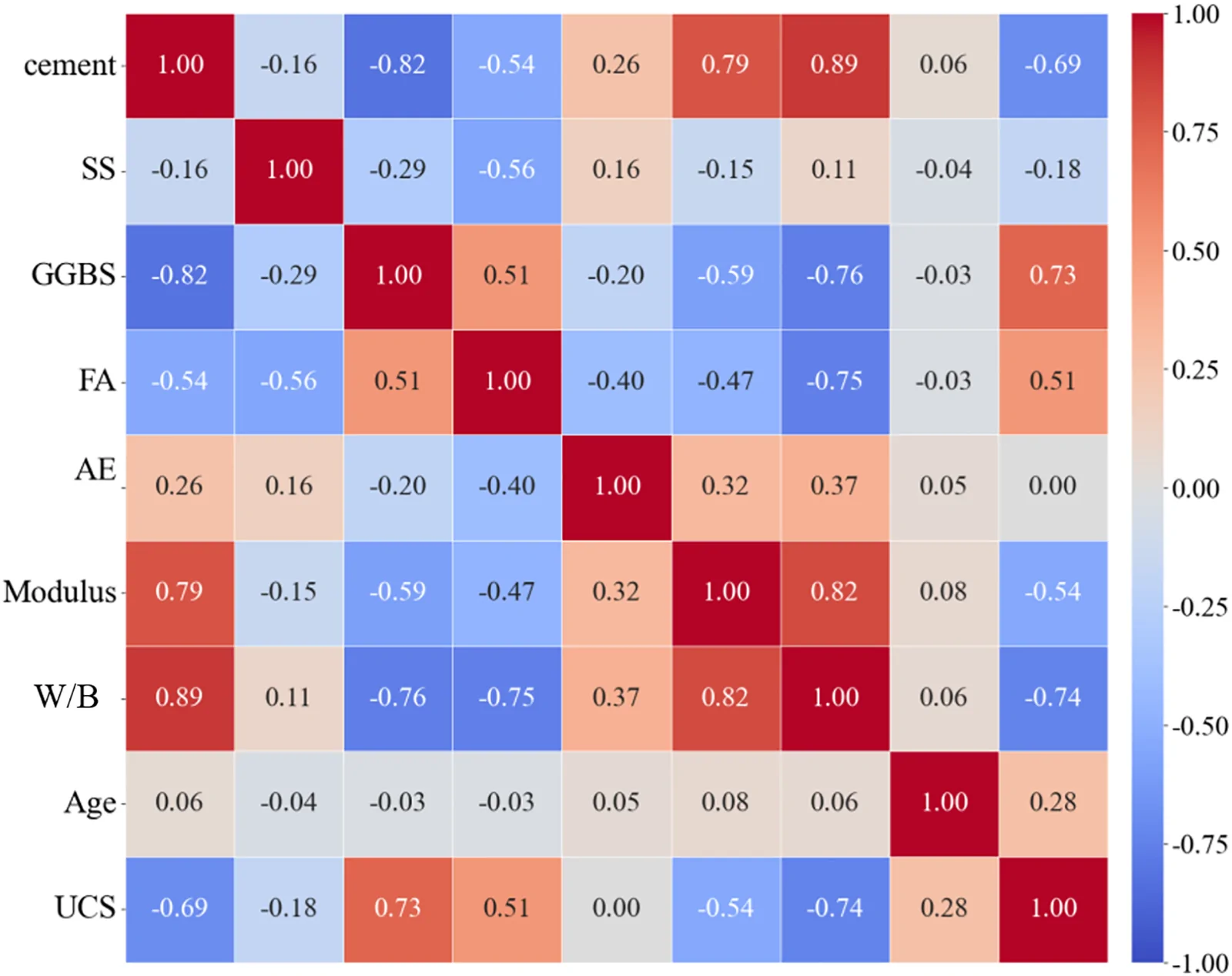
Correlation matrix illustrating the relationships among variables in the dataset.

Based on the correlation matrix analysis shown in Fig 1, a clear relationship exists between mixture composition and mechanical properties. The cement content exhibits a strong positive correlation with the water–binder ratio and alkali activator modulus (Modulus), with correlation coefficients of 0.89 and 0.79, respectively. However, it shows a pronounced negative correlation with the dosage of ground granulated blast furnace slag and with compressive strength, with correlation coefficients of −0.82 and −0.69, respectively. In contrast, the compressive strength is positively correlated with supplementary cementitious materials such as slag powder and fly ash, with correlation coefficients of 0.73 and 0.51, respectively, while exhibiting a strong negative correlation with the water–binder ratio (−0.74). These results indicate that, within this material system, cement and supplementary cementitious materials play significant but opposing roles in the development of compressive strength.

### 2.2. Generative Adversarial Networks

Goodfellow et al. [36] first proposed the framework of generative adversarial networks (GANs), originally developed to generate artificial images closely resembling the features of real photographs. A GAN consists of a generator (G) and a discriminator (D). The generator aims to accurately capture the distribution characteristics of the sample data, while the discriminator functions essentially as a binary classifier, tasked with distinguishing whether the input data originate from the original dataset or are generated by the model. The training process focuses on minimizing the distributional discrepancy between real and synthetic data. The corresponding optimization objective function is expressed as Eq. (1).


$$\begin{array}{c} \underset{G}{min}\underset{D}{max}V\left(G,D\right)=E_{x\;p_{data}\left(x\right)}\left[logD\left(x\right)\right]+E_{z\;p_{z}\left(z\right)}\left[log\left(1-D\left(G\left(z\right)\right)\right)\right] \end{array}$$ (1)

Here, *x* represents the original data, and *z* denotes the latent noise. *E*(*) indicates the expectation over a given distribution, *P*_*data*_(*x*) represents the distribution of real samples, and *P*_*z*_(*z*) is defined over a low-dimensional noise distribution. *D*(*x*) denotes the discriminator’s output for original data *x*, while *G*(*z*) represents the fake data generated by the generator from random input *z*. The discriminator aims to correctly distinguish original samples from generated samples, whereas the generator seeks to produce samples that are classified as original by the discriminator. In the non-saturating formulation, the generator is trained by maximizing log*D*(*G*(*z*)).

**Fig 2:**
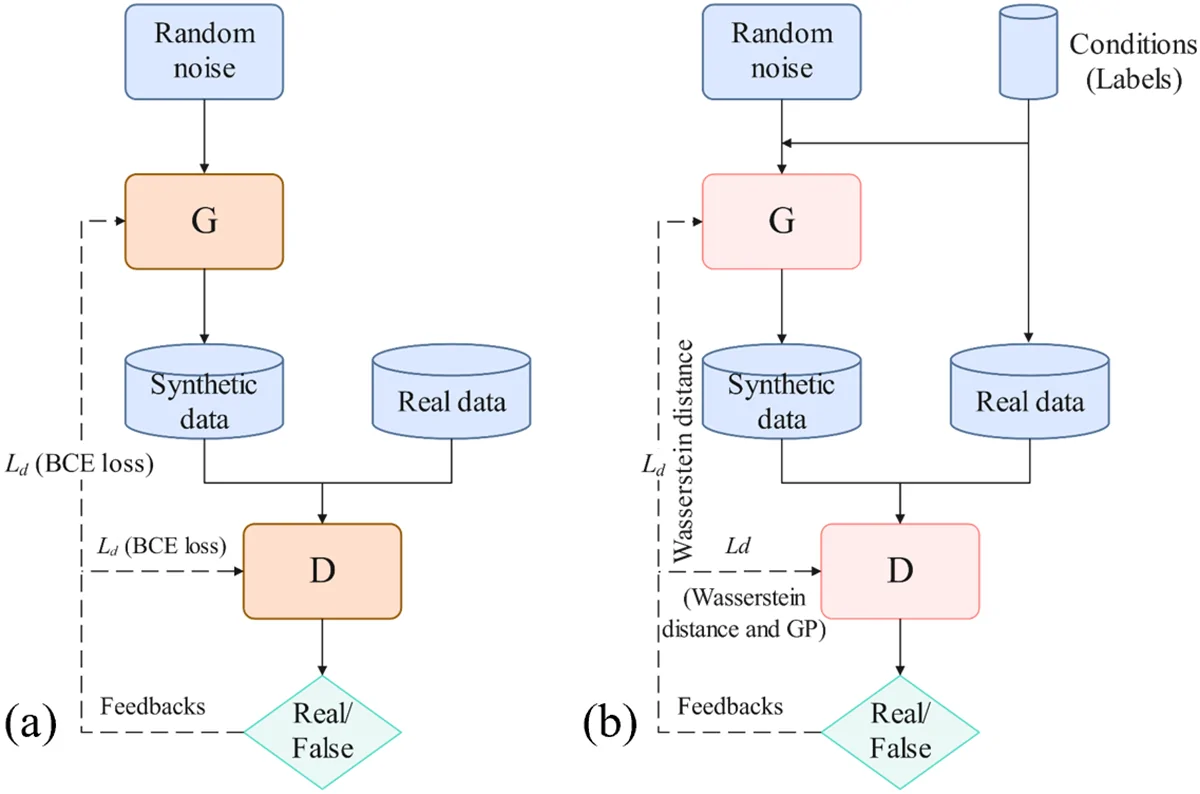
Workflow diagrams of GAN and WGAN-GP. (a) GAN. (b) WGAN-GP.

Through iterative optimization, the generator’s ability to produce realistic samples continually improves, making the generated data increasingly similar to original data and thereby increasing the difficulty for the discriminator. When the generator fully learns the probability distribution of the original data, it can generate samples that are both novel and highly faithful, effectively reproducing the statistical patterns of the original dataset. The workflow of a GAN is illustrated in Fig 2(a).

The Wasserstein GAN with Gradient Penalty (WGAN-GP) was proposed to address the instability, vanishing gradients, and slow convergence commonly encountered in traditional GANs [37]. WGAN-GP introduces the Wasserstein loss function, replacing the Jensen-Shannon (JS) divergence used in standard GANs. Unlike KL or JS divergences, the Wasserstein distance can effectively measure the difference between two distributions even when their supports do not overlap [38].

Additionally, a gradient penalty (GP) is introduced to replace the weight clipping used in the original WGAN, modifying the discriminator’s objective function as shown in Eq. (2). The gradient norm of the discriminator with respect to its input is constrained near 1 to enforce the Lipschitz continuity condition.


$$\begin{array}{c} L_{D}=E_{x\;P_{r}}\left[D\left(x\right)\right]-E_{z\;P_{z}}\left[D\left(G\left(z\right)\right)\right]+\lambda \cdot E_{\hat{x}\;P_{\hat{x}}}\left[{\left({\|\nabla_{\hat{x}}D\left(\hat{x}\right)\|}_{\text{2}}-1\right)}^{\text{2}}\right] \end{array}$$ (2)

Here, *L*_*D*_ denotes the loss function of the discriminator. $E_{x\;P_{r}}\left[D\left(x\right)\right]$ represents the expected output of the discriminator on real samples, reflecting its ability to correctly identify original data. The term $-E_{z\;P_{z}}\left[D\left(G\left(z\right)\right)\right]$ is the negative expected output of the discriminator on generated samples; together with the previous term, it corresponds to the dual form of the Wasserstein distance. The term $\lambda \cdot E_{\hat{x}\;P_{\hat{x}}}\left[{\left({\|\nabla_{\hat{x}}D\left(\hat{x}\right)\|}_{\text{2}}-1\right)}^{\text{2}}\right]$ represents the gradient penalty. The workflow of WGAN-GP is illustrated in Fig 2(b).

The WGAN-GP model was implemented using fully connected neural networks. The generator took a 128 dimensional random noise vector as input and consisted of three hidden layers, each containing 256 neurons, with ReLU activation functions. A Tanh activation function was adopted in the output layer. The discriminator also consisted of three hidden layers, each containing 256 neurons, with LeakyReLU activation functions using a negative slope of 0.2. The final output layer contained one neuron without an activation function. Both the generator and discriminator were optimized using the Adam optimizer with a learning rate of 0.0001, *β*_1_ = 0.5, and *β*_2_ = 0.999. The batch size was set to 64, the gradient penalty coefficient was 10, and the discriminator was updated five times for each generator update. The model was trained for 4000 epochs, and 2000 synthetic samples were generated for subsequent model training.

### 2.3. Machine learning models

To fully exploit the relationships between various input features and the unconfined compressive strength (UCS), this study employs multiple machine learning models to predict the UCS of the grouting materials.

#### 2.3.1. Extreme gradient boosting.

**Fig 3:**
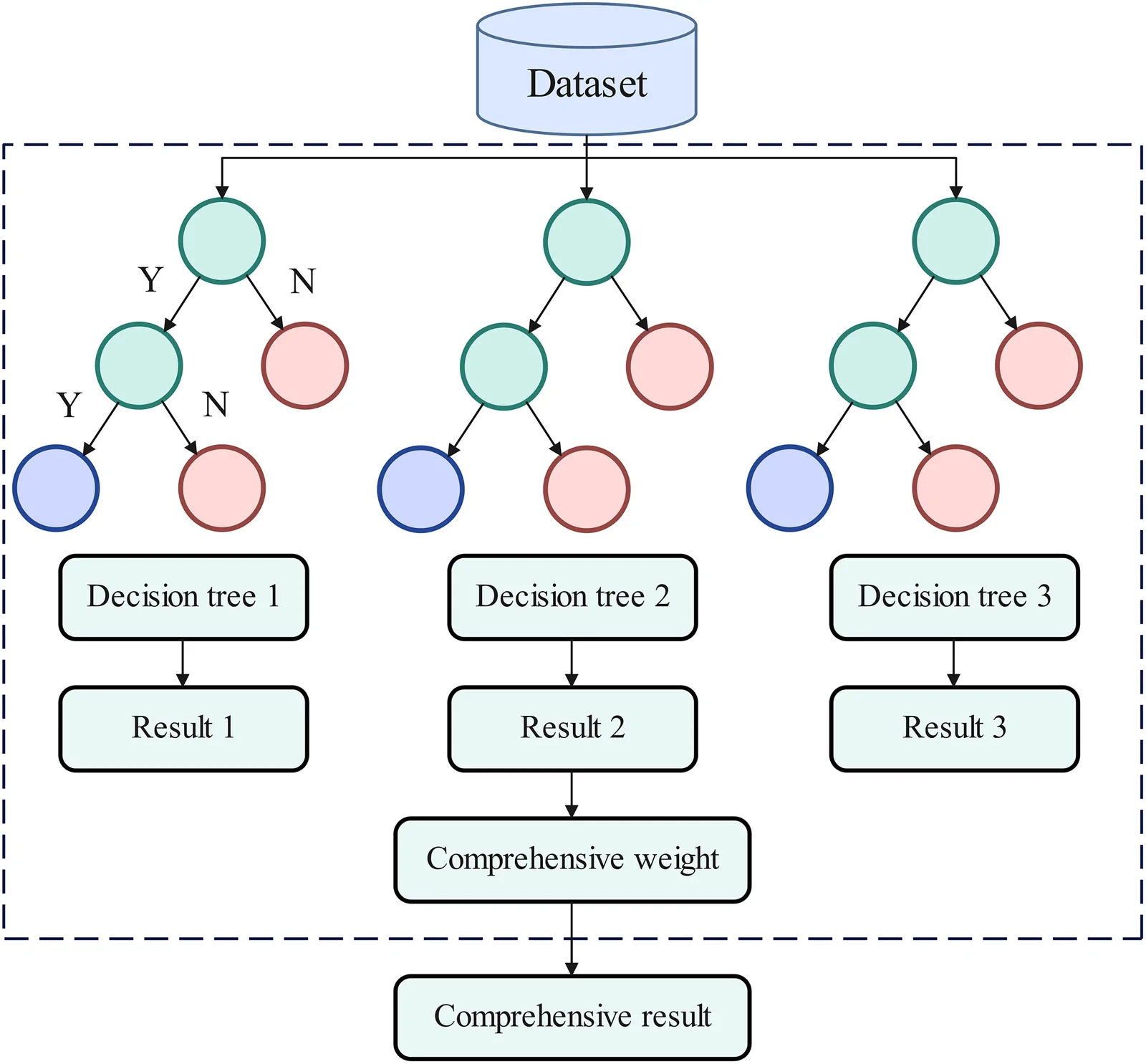
Principle of the XGBoost Algorithm.

Extreme Gradient Boosting (XGBoost) [39] is an ensemble learning method based on gradient boosting. It iteratively trains a series of weak learners and combines them in a weighted manner to form a strong predictive model, as illustrated in Fig 3.

In each iteration, XGBoost optimizes the model’s predictive capability by adjusting the model parameters to minimize the objective function. XGBoost demonstrates strong advantages when handling small datasets, effectively mitigating overfitting even with limited data. Moreover, its powerful tree-based structure and efficient training algorithm enable it to fully capture underlying patterns within the data.

The final prediction of XGBoost is expressed as the weighted sum of multiple decision trees, as shown in Eq. (3).


$$\begin{array}{c} {\hat{y}}_{i}=\sum_{k=1}^{K}f_{k}\left(x_{i}\right) \end{array}$$ (3)

Here, the input for sample *i* is *x*_*i*_, *f*(*x*_*i*_) denotes the prediction function of the *k*-th-tree, and *K* represents the total number of trees.

The core of XGBoost model training lies in minimizing an objective function that includes both a loss term and a regularization term. The loss term quantifies the discrepancy between predicted and actual values, while the regularization term constrains model complexity to prevent overfitting and favors simpler tree structures. The corresponding objective function is presented in Eqs. (4),(5).


$$\begin{array}{c} ℒ\left(\theta \right)=\sum_{i=1}^{n}ℒ\left(y_{i},{\hat{y}}_{i}\right)+\sum_{k=1}^{K}\Omega \left(f_{k}\right) \end{array}$$ (4)

$$\begin{array}{c} \Omega \left(f_{k}\right)=\gamma T+\frac{\text{1}}{\text{2}}\lambda \sum_{j=1}^{T}w_{i}^{\text{2}} \end{array}$$ (5)

Here, $ℒ\left(y_{i},{\hat{y}}_{i}\right)$ represents the loss function, $y_{i}$ is the true value of the *i*-th sample, and ${\hat{y}}_{i}$ is the predicted value. $\Omega \left(f_{k}\right)$ denotes the regularization term, $f_{k}$ refers to the *k*-th tree, *T* is the number of leaves in the tree, *W*_*j*_ is the weight of leaf, γ is the leaf count penalty, and λ is the *L*_2_ regularization coefficient.

#### 2.3.2. Newton–Raphson-Based Optimizer.

The Newton–Raphson-Based Optimizer (NRBO) is a novel intelligent optimization algorithm proposed by Sowmya et al. [40]. Inspired by the Newton–Raphson method, NRBO explores the entire search space through the Newton–Raphson Search Rule (NRSR) and the Trap Avoidance Operator (TAO), while further refining the optimal solution using multiple matrix groups. Specifically, NRSR enhances the search capability and accelerates convergence by leveraging the Newton–Raphson method to obtain superior positions within the search space. Meanwhile, TAO helps NRBO avoid local optima traps. NRBO demonstrates high efficiency, accuracy, and excellent global search performance, making it suitable for a wide range of complex optimization tasks. The optimization procedure is as follows:

(1) Determine a suitably sized random initial population, set the decision factor (DF) values, and define the maximum number of iterations.
(2) Randomly initialize the positions of the population.
(3) Combine NRSR and TAO-first, NRSR adjusts vector positions to obtain suboptimal solutions; then, based on DF, TAO determines whether to activate trap avoidance and dynamically updates the optimal position.
(4) Compare the current optimal position with that from the previous iteration, retaining only the superior solution.
(5) Repeat until the stopping criterion is met, then output the global optimal position and corresponding fitness value.

**Fig 4:**
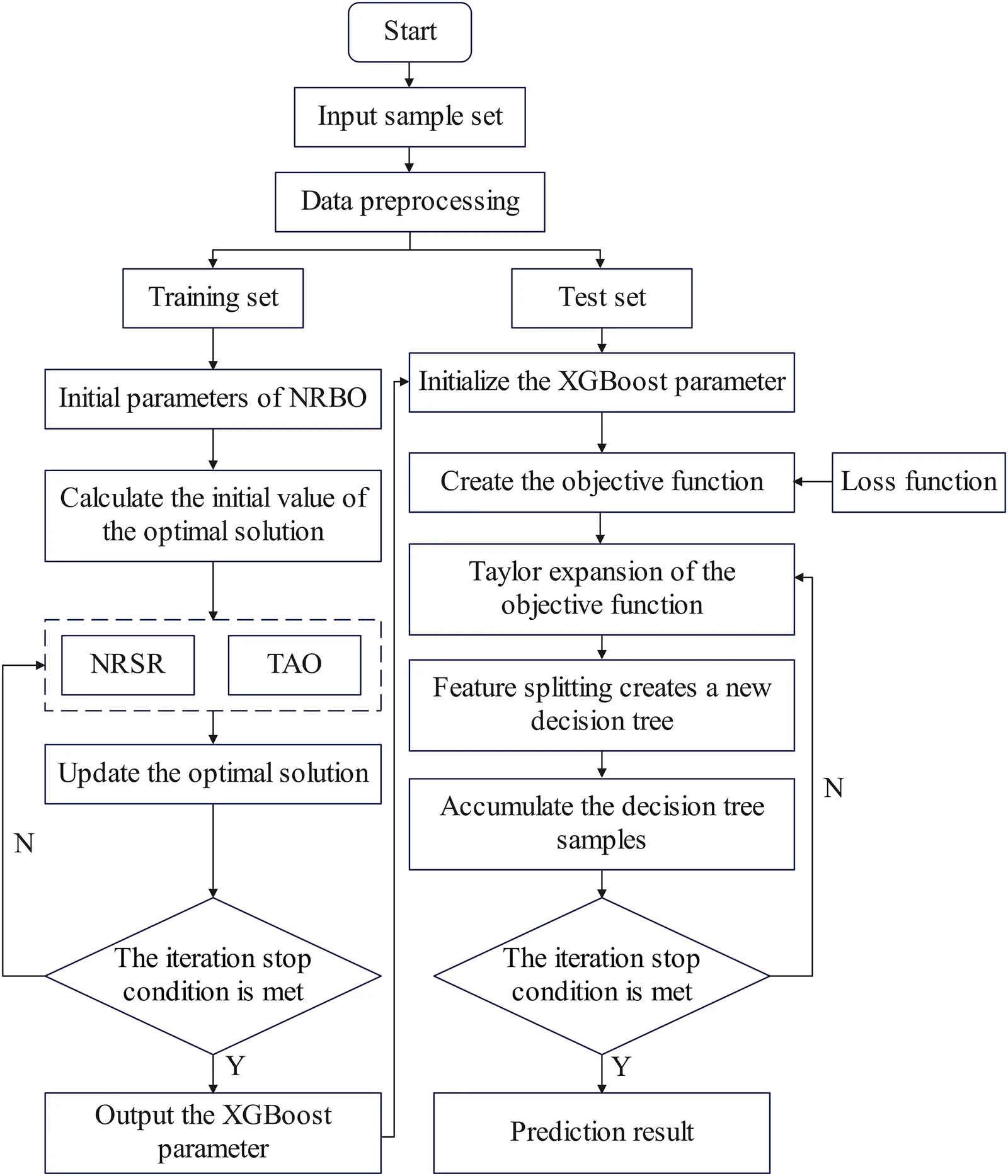
NRBO-XGBoost Algorithm Model.

As an innovative method, NRBO addresses critical gaps in high-dimensional space optimization, offering a new approach and technical pathway for solving complex real-world problems. Its combination with XGBoost, referred to as NRBO-XGBoost, is illustrated in the workflow in Fig 4.

In the NRBO-XGBoost model, five key hyperparameters of XGBoost were optimized, including the number of boosting trees, maximum tree depth, learning rate, sample subsampling ratio, and feature subsampling ratio for each tree. To ensure the reproducibility of the optimization process, the bounded search space of each hyperparameter was predefined before NRBO optimization. The search range was determined based on preliminary grid-search trials and commonly used XGBoost parameter settings, as listed in Table 2.

**Table 2:** Search space of XGBoost hyperparameters optimized by NRBO.

Hyperparameter	Description	Lower bound	Upper bound
n_estimators	Number of boosting trees	100	300
max_depth	Maximum tree depth	1	5
learning_rate	Learning rate	0.01	0.30
subsample	Sample subsampling ratio	0.50	1.00
colsample_bytree	Feature subsampling ratio for each tree	0.70	1.00

#### 2.3.3. Other machine learning models.

In this study, four additional machine learning models were selected for predicting the compressive strength of grouting materials: support vector regression (SVR), random forest (RF), Light Gradient Boosting Machine (Light GBM), and ridge regression (RR).SVR maps samples into a high-dimensional space using kernel functions and performs regression by applying the maximum margin principle under an ε-insensitive loss. RF constructs multiple decision trees via bootstrap sampling and reduces variance in predictions through averaging or majority voting. Light GBM employs an efficient histogram-based splitting method and a leaf-wise growth strategy, enabling fast processing of large-scale datasets and automatic handling of categorical features. RR extends linear regression with an L_2_ regularization term, effectively mitigating multicollinearity issues and enhancing model robustness. Together, these methods encompass a variety of modeling approaches, allowing for a comprehensive capture of the multidimensional features and complex relationships within the dataset.

#### 2.3.4. Data splitting and validation.

To evaluate the generalization capability of the proposed model, the original dataset containing 179 experimental records was first divided into a modeling dataset and an external validation dataset. Specifically, 15 independent experimental samples were reserved as an external validation set before any preprocessing, normalization, data augmentation, hyperparameter optimization, or model training. The remaining 164 original samples were used for WGAN-GP-based data generation and subsequent machine learning model development.

To avoid data leakage, the external validation set was completely isolated from the data generation and model training process. The normalization parameters were estimated only from the 164 modeling samples and were then applied to the external validation samples. The WGAN-GP model was trained exclusively using the 164 modeling samples, and the 15 external validation samples were not involved in synthetic data generation, hyperparameter optimization, or model fitting.

After WGAN-GP training, 2000 synthetic samples were generated and combined with the 164 original modeling samples to form an augmented dataset containing 2164 samples. This augmented dataset was then used for machine learning model training and hyperparameter optimization. During model development, five-fold cross-validation was performed within the augmented modeling dataset to tune hyperparameters and reduce the risk of overfitting. Finally, the predictive performance and generalization capability of the developed model were evaluated using the 15 independent external validation samples, which contained only original experimental data. This strategy ensured that the validation samples were completely unseen during data augmentation and model development, thereby preventing information from the external validation set from entering the data augmentation and model-development procedures. To ensure reproducibility, all experiments were implemented in Python 3.11.0. The generated samples, data split indices, and model scripts were saved to facilitate reproducibility.

#### 2.3.5. Performance evaluation metrics.

In this study, three well-established performance evaluation metrics—the coefficient of determination (R^2^), mean absolute error (MAE), and root mean square error (RMSE)—were adopted to comprehensively quantify the predictive performance of the regression models. Specifically, R^2^ measures the model’s ability to explain the variance of the dependent variable. MAE reflects the average absolute deviation between the predicted and observed values, providing an intuitive measure of the overall magnitude of prediction errors. RMSE, obtained by taking the square root of the mean squared error, amplifies the influence of larger errors and is therefore more sensitive to extreme prediction deviations. These three metrics complement each other from different perspectives, enabling a comprehensive assessment of model accuracy, stability, and reliability. The corresponding formulations are given in Eqs. (6)–(8).


$$\begin{array}{c} R^{\text{2}}=1-\frac{\sum_{i=1}^{N}{\left(y_{i}-{\hat{y}}_{i}\right)}^{\text{2}}}{\sum_{i=1}^{N}{\left(y_{i}-{\overset{―}{y}}_{i}\right)}^{\text{2}}} \end{array}$$ (6)

$$\begin{array}{c} \text{MAE}=\frac{\text{1}}{N}\sum_{i=1}^{N}\left|y_{i}-{\hat{y}}_{i}\right| \end{array}$$ (7)

$$\begin{array}{c} \text{RMSE}=\sqrt{\frac{\text{1}}{N}\sum_{i=1}^{N}{\left(y_{i}-{\hat{y}}_{i}\right)}^{\text{2}}} \end{array}$$ (8)

where *N* is the number of samples; $y_{i}$ denotes the measured UCS; ${\hat{y}}_{i}$ represents the predicted UCS; and ${\overset{―}{y}}_{i}$ is the mean value of UCS.

#### 2.3.6. SHAP analysis.

Although nonlinear machine learning models perform well in tabular regression tasks, they often lack interpretability [41]. The Shapley Additive Explanations model (SHAP), grounded in game theory, provides a post hoc interpretability technique [42]. This approach evaluates the marginal contribution of each feature to the model output. By applying the SHAP method, precise contribution values are computed and aggregated to explain the predicted output of the model, as defined in Eqs. (9)–(11). The SHAP framework quantifies both the influence and importance of each feature while examining the patterns through which these features affect the predictions. Moreover, it enables in-depth analysis of feature impacts at the individual sample level, including their positive and negative contributions, thereby facilitating both global and local interpretability.


$$\begin{array}{c} g\left(x\right)=\varphi_{\text{0}}+\sum_{j=1}^{M}\varphi_{j}\cdot x_{j}=f\left(x\right) \end{array}$$ (9)

$$\begin{array}{c} \varphi_{j}=\sum_{S}\frac{\left|S\right|!(n-|S|-1)!}{M!}\left(f_{x}\left(S\cup \left\{x_{j}\right\}\right)-f_{x}\left(S\right)\right) \end{array}$$ (10)

$$\begin{array}{c} S\subseteq \left\{x_{\text{1}},x_{\text{2}},\ldots x_{p}\right\}/\left\{x_{j}\right\} \end{array}$$ (11)

## 3. Results and analysis

### 3.1. WGAN-GP data generation

**Fig 5:**
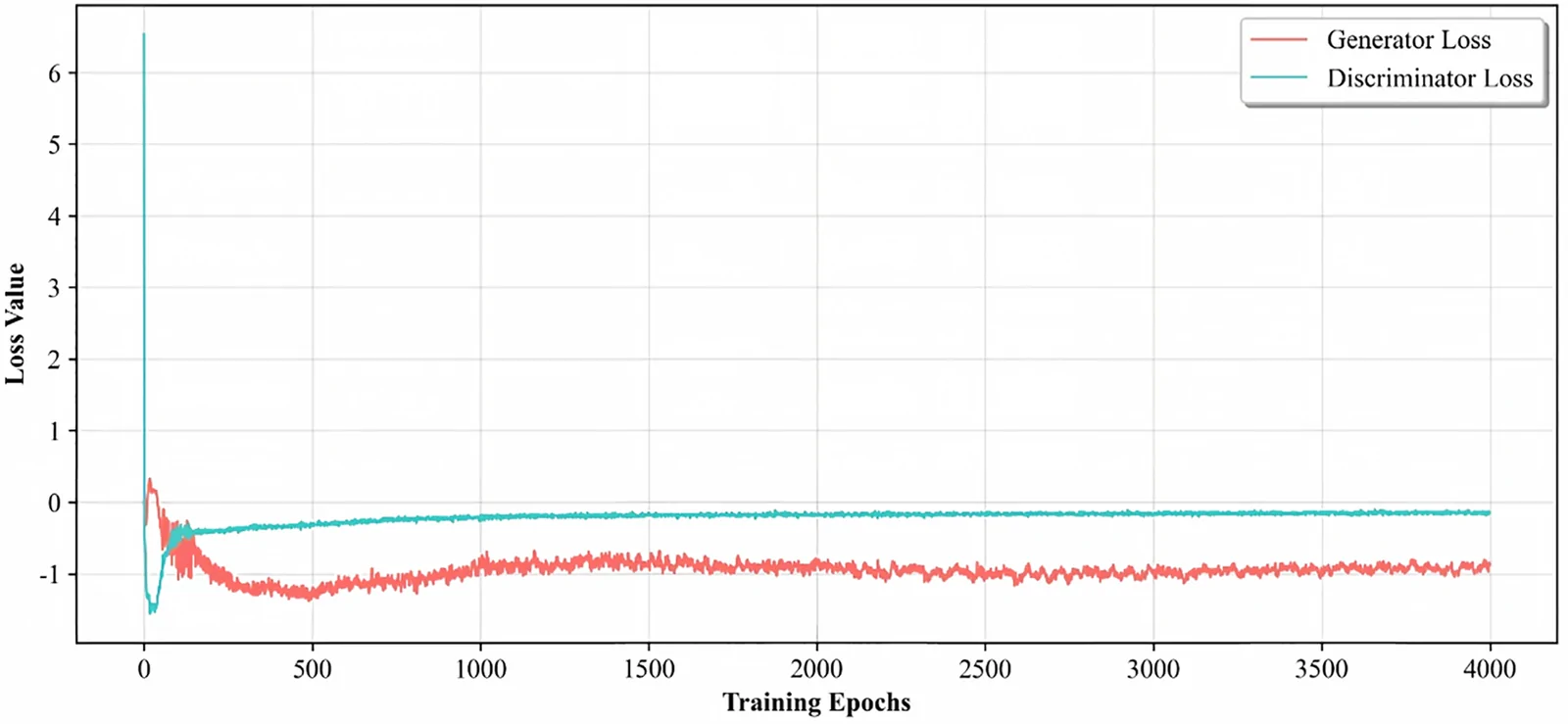
Loss of the WGAN-GP Model.

Fig 5 illustrates the variation of the generator loss and discriminator loss of the WGAN-GP model over training epochs. In the early stages of training, both losses exhibit noticeable fluctuations; however, as the number of epochs increases (around 500 epochs), the loss curves gradually stabilize and oscillate slightly near zero. This behavior intuitively indicates that the adversarial process between the generator and discriminator has reached a dynamic equilibrium, reflecting a stable training state. According to the experimental setup, the total number of training epochs (4000) was determined based on the requirement to optimize the quality of the generated data. Further increasing the number of epochs may lead to training instability, which could degrade the distributional fidelity of the generated data.

**Fig 6:**
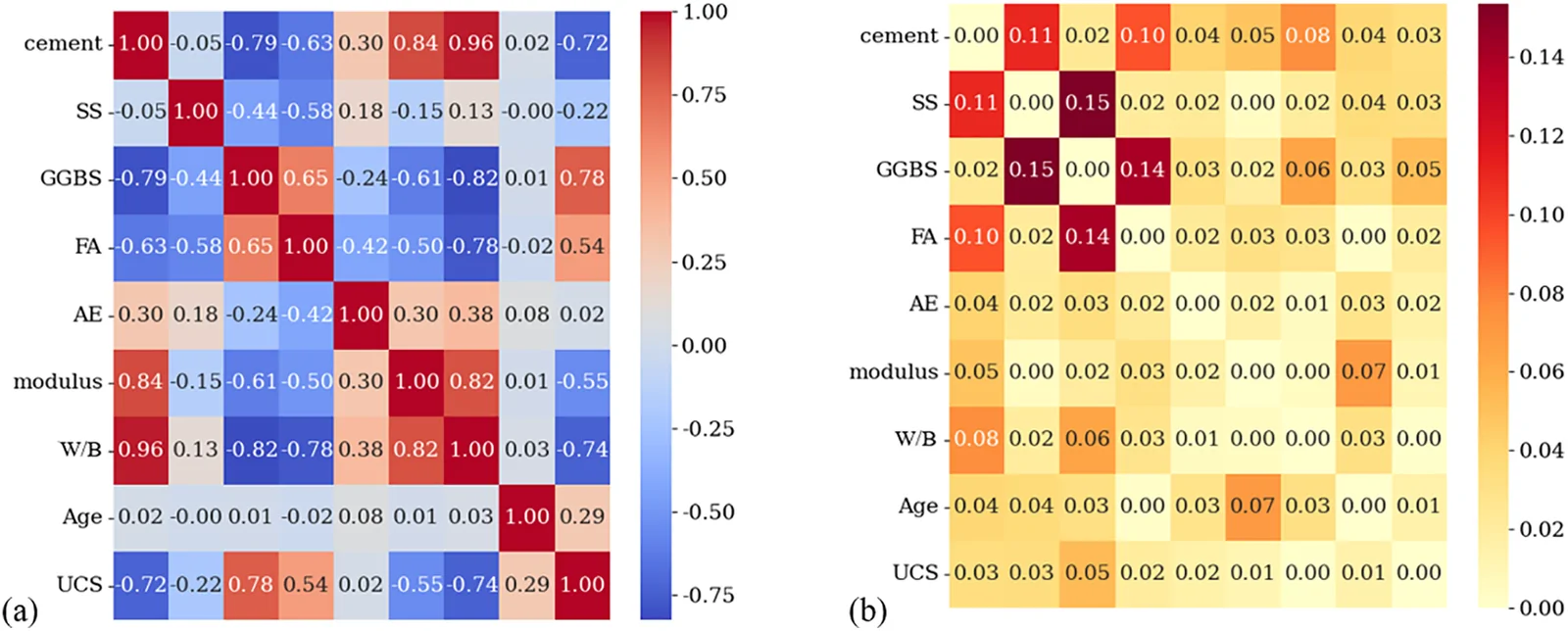
Correlation heatmaps and correlation differences between the original and WGAN-GP-generated datasets. (a) Correlation matrix of generated data. (b) Correlation difference.

By analyzing the correlation heatmap of the original data in Fig 1 alongside the correlation heatmap of the generated data in Fig 6, several observations can be made. The correlation coefficient heatmap of the generated dataset in Fig 6(a) shows that the strength of associations among features closely matches the correlation patterns of the corresponding features in the original dataset, visually demonstrating the similarity in variable relationships between the generated and original data. Fig 6(b), which presents the heatmap of correlation coefficient differences, further quantifies the deviations between the two datasets. The mean difference is only 0.0349, and most feature pairs exhibit differences below 0.05, indicating that the deviation in feature correlation structure between the generated and original datasets is minimal. These results quantitatively confirm the consistency of the generated data’s distributional characteristics with those of the original data and indirectly demonstrate that the generative model effectively captures the underlying distribution of the original dataset.

To further evaluate the statistical reliability and physical plausibility of the WGAN-GP-generated samples, the statistical characteristics of the original and generated datasets were compared, as shown in Table 3. The results indicate that the generated data are generally consistent with the original experimental data in terms of minimum, maximum, mean, and standard deviation. For the input variables, the mean differences are relatively small. In particular, the mean differences of GGBS, FA, AE, Modulus, and W/B are 0.08, 0.11, 0.04, 0.01, and 0.00, respectively, indicating that the generated samples effectively preserve the central tendency of the original dataset. The standard deviation differences of most variables are also limited, suggesting that the dispersion characteristics of the original experimental samples are reasonably maintained.

**Table 3:** Statistical characteristics and absolute differences between the original and WGAN-GP generated datasets.

Statistic	Cement	SS	GGBS	FA	AE	Modulus	W/B	Age	UCS
Min-Original	0.00	0.00	0.00	0.00	2.00	0.80	0.32	1.00	2.20
Min-Generated	0.00	0.00	0.00	0.00	2.00	0.87	0.32	1.02	2.29
Min-Difference	0.00	0.00	0.00	0.00	0.00	0.07	0.00	0.02	0.09
Max-Original	100.00	60.00	100.00	40.00	12.00	1.80	0.80	28.00	86.20
Max-Generated	100.00	58.49	97.26	39.73	11.95	1.80	0.80	28.00	80.14
Max-Difference	0.00	1.51	2.74	0.27	0.05	0.00	0.00	0.00	6.06
Mean-Original	24.58	17.21	52.18	6.03	6.30	1.46	0.64	13.28	27.53
Mean-Generated	24.16	17.46	52.10	6.14	6.26	1.47	0.64	12.59	26.07
Mean-Difference	0.42	0.25	0.08	0.11	0.04	0.01	0.00	0.69	1.46
Std-Original	22.72	15.32	16.70	12.20	1.81	0.24	0.20	11.07	18.31
Std-Generated	21.72	15.40	16.87	12.22	1.82	0.24	0.19	10.52	16.95
Std-Difference	0.99	0.08	0.17	0.02	0.01	0.00	0.00	0.55	1.36

In terms of variable ranges, all generated samples remain within or very close to the experimentally observed ranges. No negative binder contents, unrealistic alkali equivalent values, excessive activator modulus values, or invalid strength values were produced. Although the maximum UCS value of the generated dataset is slightly lower than that of the original dataset, with an absolute difference of 6.06 MPa, the generated UCS values still remain within the original experimental range. This indicates that the WGAN-GP model tends to provide a conservative representation of the high-strength region rather than generating physically unrealistic extreme values. Therefore, the generated dataset can be considered statistically consistent and physically plausible for subsequent model training.

**Fig 7:**
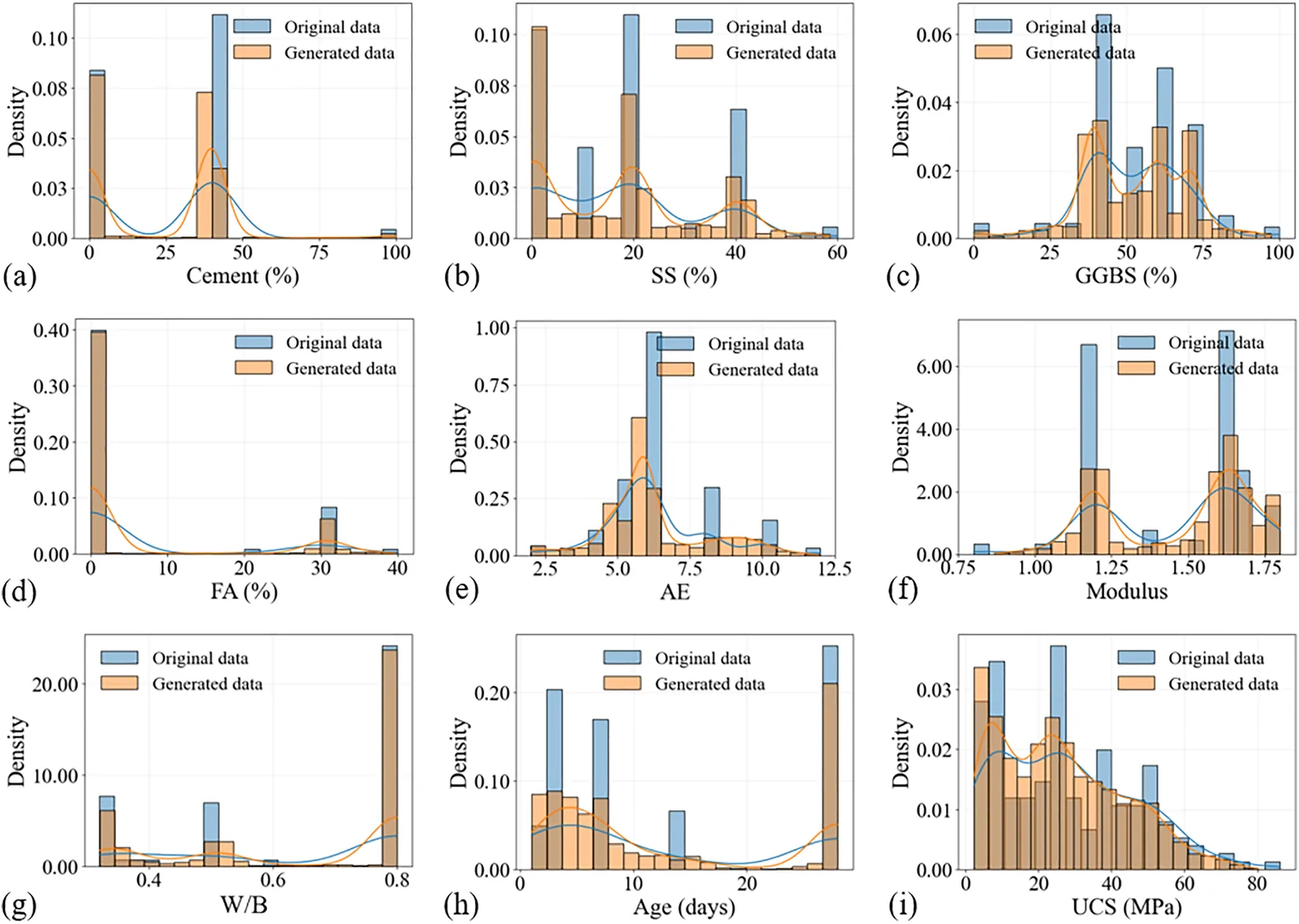
Comparison of marginal distributions between the original and WGAN-GP-generated datasets for all variables. (a) Cement. (b) SS. (c) GGBS. (d) FA. (e) AE. (f) Modulus. (g) W/B. (h) Age. (i) UCS.

In addition to the statistical comparison presented in Table 3, histograms and kernel density estimation (KDE) curves were used to further evaluate the distributional consistency between the original and WGAN-GP-generated datasets, as shown in Fig 7. Overall, the generated samples exhibit distributional patterns similar to those of the original experimental data for most variables, including Cement, SS, GGBS, FA, AE, Modulus, W/B, Age, and UCS. The generated data generally cover the main value ranges of the original dataset and reproduce the major distribution peaks.

For mixture proportion variables, such as Cement, SS, GGBS, and FA, the generated data retain the main concentration regions observed in the original samples, indicating that the WGAN-GP model effectively captures the compositional characteristics of the experimental mixtures. For AE, Modulus, W/B, and Age, the generated distributions are also generally consistent with the original data, suggesting that the generated samples remain within reasonable parameter ranges. Moreover, the UCS distribution of the generated data follows the overall trend of the original UCS distribution, without producing negative or excessively high strength values.

Although slight deviations can be observed in some local density peaks, especially for variables with discrete or highly concentrated experimental values, these differences are acceptable considering the limited size and discrete nature of the original dataset. Overall, the histogram and KDE comparisons demonstrate that the WGAN-GP-generated dataset preserves the marginal distribution characteristics of the original experimental data and provides statistically reasonable samples for subsequent machine learning model training.

### 3.2. Validation of the optimal model

#### 3.2.1. Hyperparameter sensitivity analysis.

To assess the robustness of the optimized XGBoost model, a one-factor-at-a-time hyperparameter sensitivity analysis was conducted. Five key hyperparameters were examined: n_estimators, max_depth, learning_rate, subsample, and colsample_bytree, with ranges of 100–300, 1–5, 0.01–0.30, 0.50–1.00, and 0.70–1.00, respectively. For each setting, five-fold cross-validation was performed, and the mean and standard deviation of R^2^ were calculated for both the original and augmented datasets.

**Fig 8:**
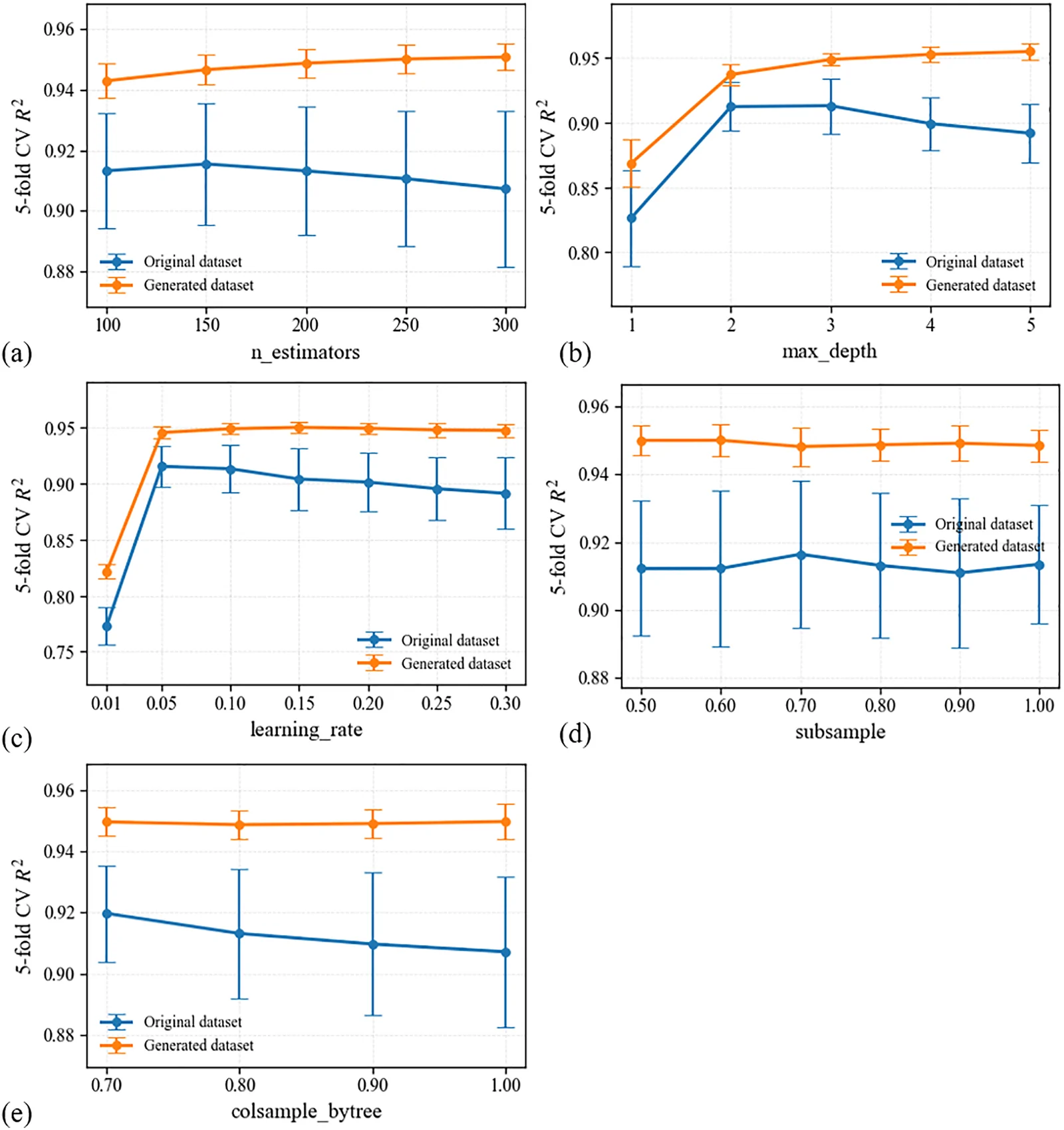
Hyperparameter sensitivity analysis of XGBoost based on five-fold cross-validation R^2^. (a) n_estimators. (b) max_depth. (c) learning_rate. (d) subsample. (e) colsample_bytree.

As shown in Fig 8, the augmented dataset generally achieved higher and more stable cross-validation R^2^ values than the original dataset. The model was more sensitive to max_depth and learning_rate, while subsample and colsample_bytree showed relatively minor effects. Overall, the results indicate that the XGBoost model maintained stable performance within the selected hyperparameter ranges, confirming the robustness of the proposed modeling framework and the reliability of the selected hyperparameter configuration.

#### 3.2.2. Validation of the NRBO-XGBoost model.

In this study, the hyperparameters of the XGBoost model were first tuned using a grid search algorithm combined with five-fold cross-validation, enabling a comparative validation of models trained on both the original and augmented datasets. Subsequently, the NRBO algorithm was applied to further optimize the XGBoost hyperparameters, identifying the most suitable configuration to enhance model performance. The optimal hyperparameter values for the baseline XGBoost model are listed in Table 4.

**Table 4:** Optimal hyperparameter tuning of XGBoost.

Model	Original Data		Augmented dataset	
	Hyperparameters	Optimal Values	Hyperparameters	Optimal Values
XGBoost	n_estimators	100	n_estimators	200
	max_depth	3	max_depth	5
	learning_rate	0.1	learning_rate	0.1
	subsample	0.8	subsample	0.8
	colsample_bytree	0.7	colsample_bytree	0.7
NRBO-XGBoost	n_estimators	180	n_estimators	232
	max_depth	2	max_depth	3
	learning_rate	0.121	learning_rate	0.148
	subsample	0.5	subsample	0.643
	colsample_bytree	0.7	colsample_bytree	1.0

**Fig 9:**
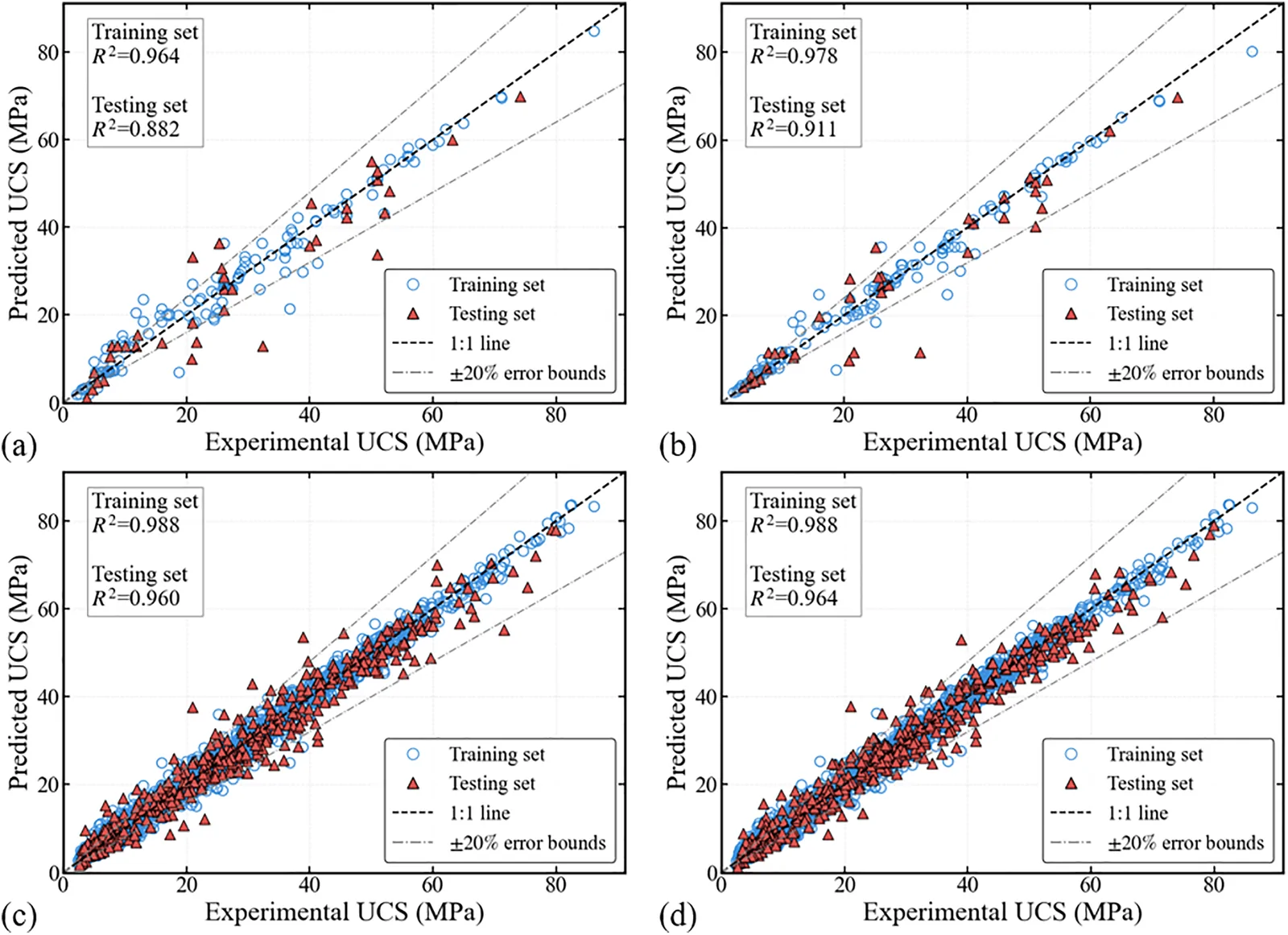
Comparison of actual and predicted values. (a) XGBoost using the original dataset. (b) NRBO-XGBoost using the original dataset. (c) XGBoost using the augmented dataset. (d) NRBO-XGBoost using the augmented dataset.

To evaluate the effectiveness of the proposed WGAN-GP and NRBO-XGBoost models in improving prediction accuracy and model reliability, a comparative analysis was conducted on the correspondence between predicted values and measured UCS under different data conditions and parameter optimization strategies. As shown in Fig 9, the line (y = x) represents the ideal fit, with a ± 20% error band introduced as an engineering criterion to assess the practical applicability of the model predictions.

For the baseline XGBoost model, the use of the augmented dataset improved the training set R^2^ from 0.964 to 0.978 and the testing-set R^2^ from 0.882 to 0.911. Compared with the model developed using the original dataset, the prediction points obtained using the augmented dataset were more concentrated around the 1:1 line, particularly in the medium- and high-strength ranges. This result indicates that WGAN-GP-based data augmentation improved the model’s ability to capture the nonlinear relationship between mixture parameters and UCS.

The application of NRBO further enhanced the predictive performance of XGBoost. Under the original-data condition, NRBO-XGBoost achieved R^2^ values of 0.988 and 0.960 for the training and testing sets, respectively, which were higher than those obtained using the baseline XGBoost model. Under the augmented-data condition, the corresponding R^2^ values reached 0.988 and 0.964. The smaller difference between the training- and testing set R^2^ values for NRBO-XGBoost also indicates a better balance between fitting accuracy and generalization performance.

Among the four modeling schemes, NRBO-XGBoost trained using the augmented dataset exhibited the best overall predictive performance. Its prediction points were distributed most closely around the 1:1 line, and only a limited number of samples showed noticeable deviations from the ± 20% error bounds. These results demonstrate that WGAN-GP data augmentation and NRBO hyperparameter optimization provide complementary improvements: data augmentation enriches the available feature distribution, whereas NRBO identifies a more suitable hyperparameter configuration for XGBoost. Their combined application therefore improves the accuracy, stability, and generalization capability of UCS prediction.

Table 5 compares the predictive performance of XGBoost and NRBO-XGBoost under the original- and augmented-data conditions. Data augmentation improved the testing R^2^ of XGBoost from 0.882 to 0.911, while reducing RMSE from 6.482 to 5.615 MPa and MAE from 4.771 to 3.627 MPa. NRBO optimization further enhanced model performance, with the testing R^2^ reaching 0.960 for the original dataset and 0.964 for the augmented dataset. The NRBO-XGBoost model trained on the augmented dataset achieved the lowest testing RMSE and MAE values of 3.411 and 2.474 MPa, respectively, demonstrating the combined effectiveness of data augmentation and hyperparameter optimization in improving prediction accuracy and generalization.

**Table 5:** Predictive performance of XGBoost and NRBO-XGBoost models.

Model	Data	Training set			Testing set		
		R^2^	RMSE	MAE	R^2^	RMSE	MAE
XGBoost	Original Data	0.964	3.444	2.277	0.882	6.482	4.771
	Augmented dataset	0.978	2.679	1.571	0.911	5.615	3.627
NRBO-XGBoost	Original Data	0.988	1.913	1.414	0.960	3.602	2.543
	Augmented dataset	0.988	1.892	1.393	0.964	3.411	2.474

#### 3.2.3. Validation.

To validate the model, a small portion of the original experimental data was reserved. These data points were separated from the original dataset prior to data generation and were not used in any part of the model development. During WGAN-GP training and new data generation, as well as UCS prediction using the NRBO-XGBoost model, these entries were entirely excluded. The validation set consists of 15 UCS test data points, all of which were completely removed from the original dataset, not involved in any simulation, and obtained through independent experimental investigation.

**Fig 10:**
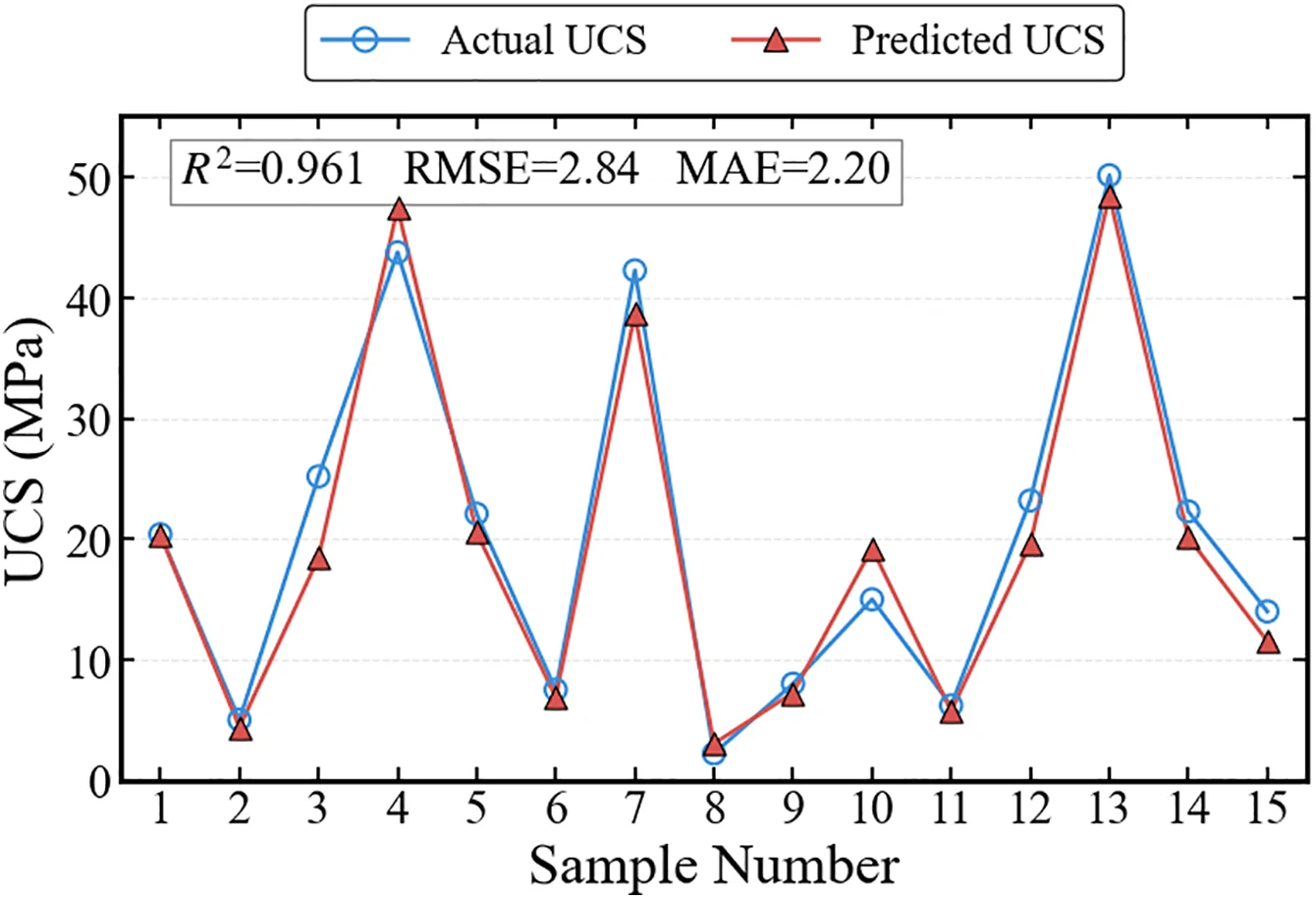
Comparison between the measured and predicted UCS values for the independent external validation dataset.

As shown in Fig 10, the comparison between the measured UCS values and the NRBO-XGBoost model predictions on the validation set is presented. The predicted curve closely follows the overall trend of the measured values, indicating that the model effectively captures the fluctuations of UCS across different samples. Although a few individual points show minor deviations, the predictions are generally distributed around the actual values, with no systematic overestimation or underestimation observed. Quantitatively, the model achieved an R^2^ of 0.961, an RMSE of 2.84 MPa, and an MAE of 2.20 MPa on the validation set, demonstrating that the prediction accuracy remains high even on a completely independent dataset. These results further confirm that the WGAN-GP-augmented dataset combined with the NRBO-XGBoost model does not rely on overfitting the original experimental data and possesses strong generalization capability.

#### 3.2.4. Other models.

**Fig 11:**
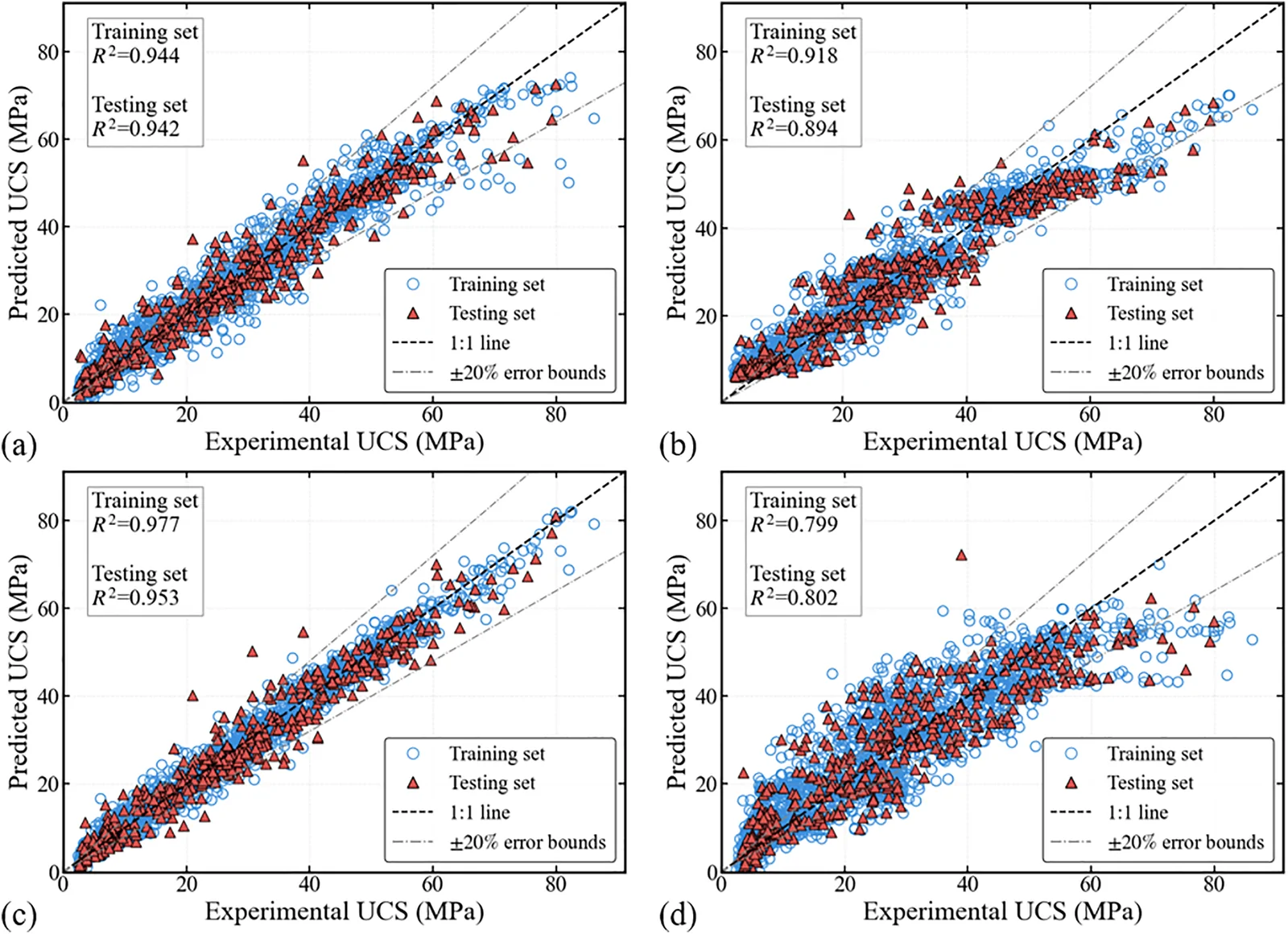
Comparison of actual and predicted values for different machine learning models. (a) SVR. (b) RF. (c) LightGBM. (d) RR.

To further validate the effectiveness and superiority of the proposed NRBO-XGBoost model, its predictive performance was compared with several commonly used machine learning models, including Support Vector Regression (SVR), Random Forest (RF), LightGBM, and Ridge Regression (RR). These models were selected for comparison due to their widespread application in tabular data modeling and engineering parameter prediction, making them representative benchmarks. It should be noted that the comparison in this section was conducted under the augmented-data condition. Specifically, the original experimental records and WGAN-GP generated samples were combined to construct an expanded dataset, and all benchmark models were trained and tested using the same data-splitting strategy. Therefore, SVR, RF, LightGBM, RR, and NRBO-XGBoost were compared under identical data conditions and evaluation procedures. Fig 11 compares the experimental and predicted UCS values obtained using SVR, RF, LightGBM, and RR. Most predictions from SVR and LightGBM are distributed close to the 1:1 line, with testing R^2^ values of 0.942 and 0.953, respectively, indicating strong predictive accuracy and generalization. RF achieved moderate performance, with a testing R^2^ of 0.894, whereas greater dispersion was observed in the medium- and high-strength ranges. RR exhibited the weakest nonlinear fitting capability, yielding a testing R^2^ of only 0.802 and noticeably larger deviations from the ideal line. Overall, LightGBM showed the best performance among the four benchmark models, followed by SVR, RF, and RR.

By combining the results in Table 5 and Table 6, a more systematic analysis of the predictive performance of different models can be conducted. The use of augmented dataset improved the testing performance of XGBoost, increasing R^2^ from 0.882 to 0.911 while reducing RMSE and MAE from 6.482 and 4.771 MPa to 5.615 and 3.627 MPa, respectively. NRBO optimization produced a further improvement, with NRBO-XGBoost trained on the augmented dataset achieving a testing R^2^ of 0.964, an RMSE of 3.411 MPa, and an MAE of 2.474 MPa. Among the common machine learning models, LightGBM exhibited the best performance, attaining a testing R^2^ of 0.953, followed by SVR, RF, and RR. Overall, NRBO-XGBoost outperformed all benchmark models in terms of predictive accuracy and error control, demonstrating the effectiveness of combining augmented dataset with NRBO-based hyperparameter optimization.

**Table 6:** Predictive performance of common machine learning models.

Model	Training set			Testing set		
	R^2^	RMSE	MAE	R^2^	RMSE	MAE
SVR	0.944	4.043	2.720	0.942	4.342	3.043
RF	0.918	4.901	3.719	0.894	5.861	4.544
LightGBM	0.977	2.582	1.905	0.953	3.905	2.838
RR	0.799	7.685	5.856	0.802	7.995	6.118

**Fig 12:**
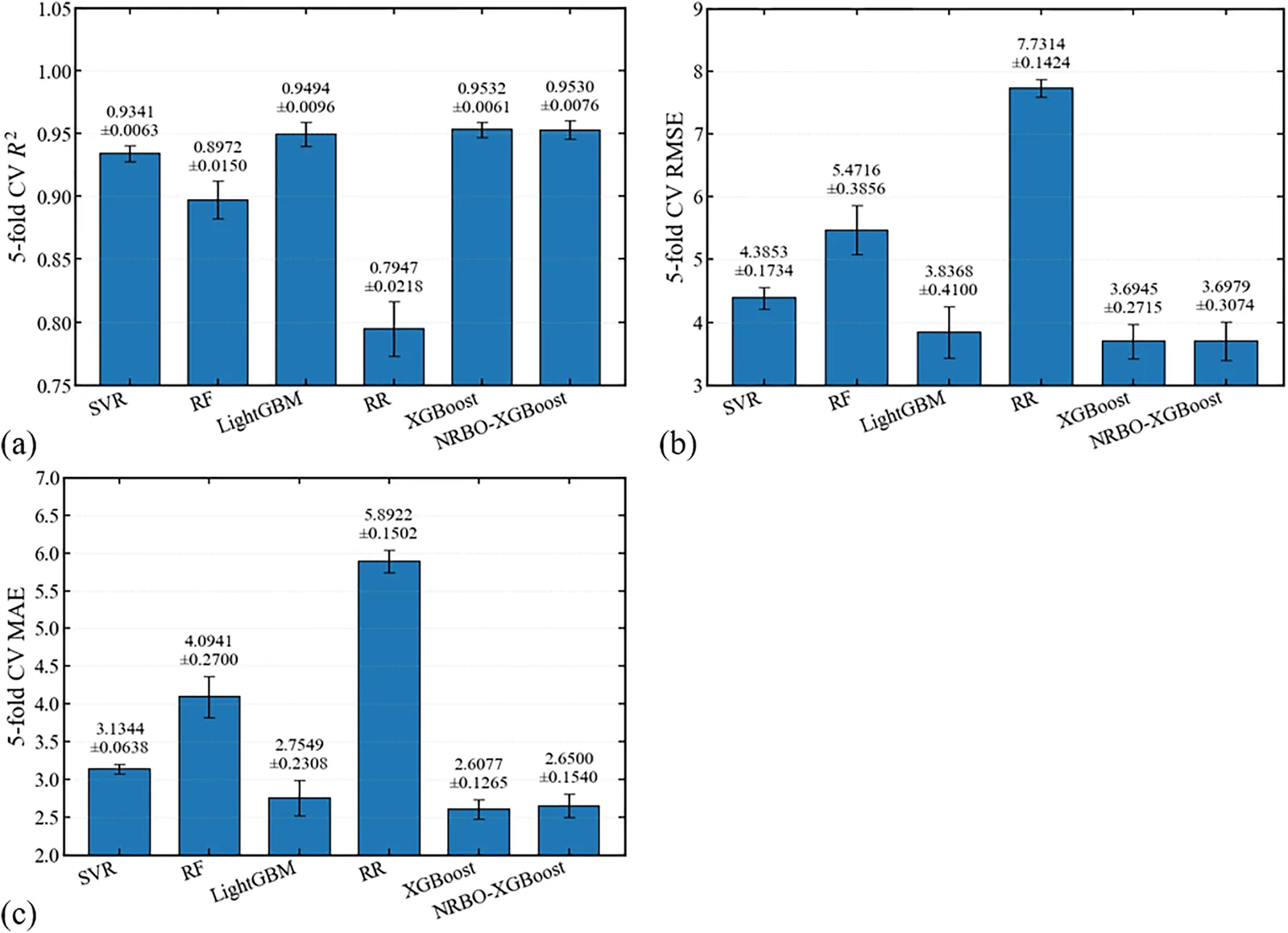
Comparison of five-fold cross-validation performance among different machine learning models. (a) R^2^. (b) RMSE. (c) MAE.

As shown in Fig 12, the six models exhibited clear differences in five-fold cross-validation performance. XGBoost and NRBO-XGBoost achieved the highest and nearly identical mean R^2^ values of 0.9532 and 0.9530, respectively, together with relatively low RMSE and MAE values. LightGBM also showed competitive performance, with a mean R^2^ of 0.9494. In contrast, RF and RR produced lower R^2^ values and larger prediction errors, particularly RR, which exhibited the weakest overall performance. The relatively small standard deviations of XGBoost and NRBO-XGBoost indicate stable performance across different folds. Overall, the cross-validation results confirm the robustness of the tree-based ensemble models, while XGBoost and NRBO-XGBoost demonstrated comparable generalization capability.

**Fig 13:**
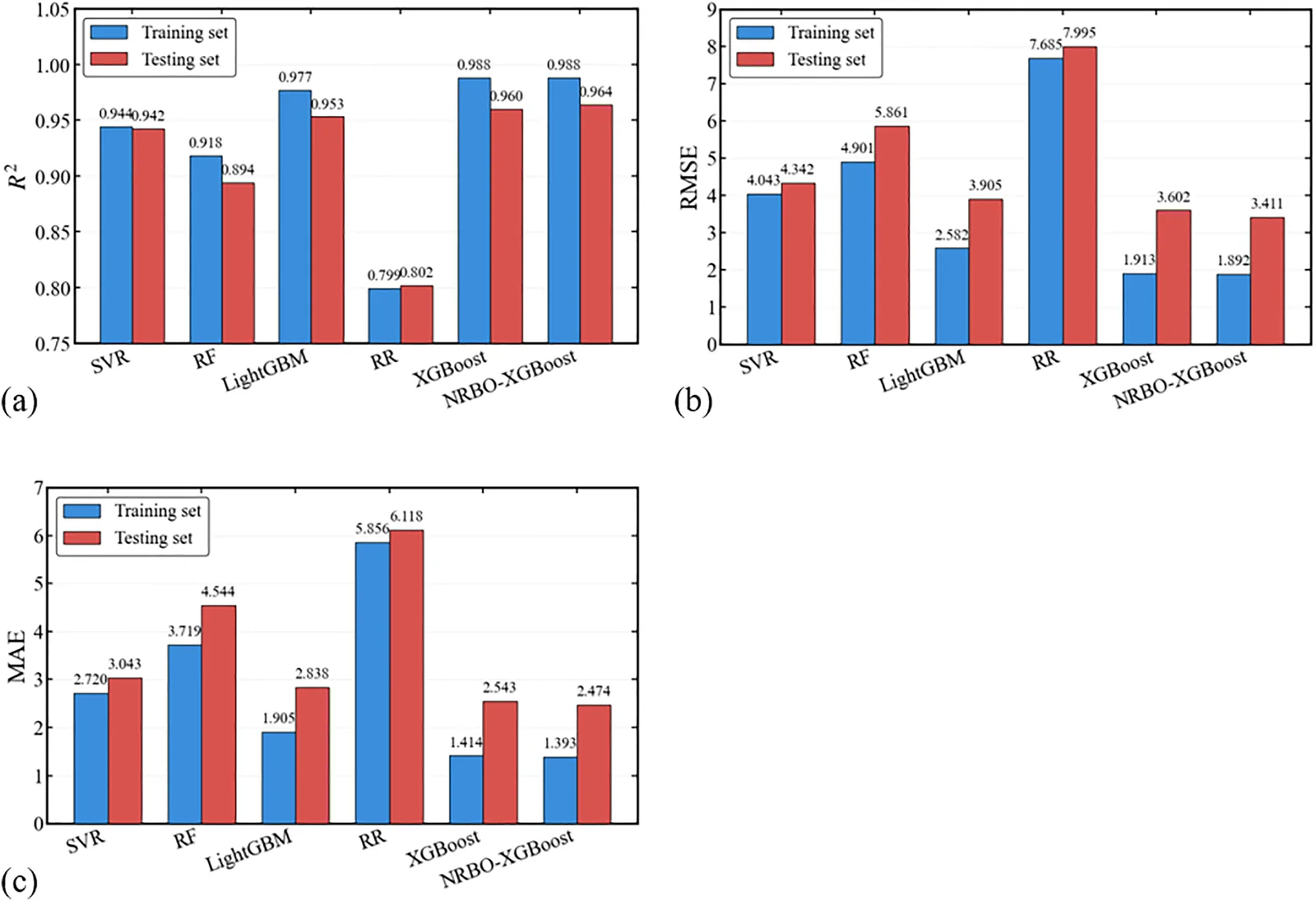
Comparison of evaluation metrics among different machine learning models. (a) R^2^. (b) RMSE. (c) MAE.

As shown in Fig 13, clear differences were observed in the predictive performance of the six machine learning models. NRBO-XGBoost achieved the best overall performance, with training and testing R^2^ values of 0.988 and 0.964, respectively, together with the lowest testing RMSE and MAE values of 3.411 and 2.474 MPa. Compared with the baseline XGBoost model, NRBO optimization slightly increased the testing R^2^ from 0.960 to 0.964 and reduced RMSE and MAE from 3.602 and 2.543 MPa to 3.411 and 2.474 MPa, respectively. LightGBM ranked second among the benchmark models, achieving a testing R^2^ of 0.953, whereas SVR also exhibited satisfactory generalization with a testing R^2^ of 0.942. RF showed moderate predictive performance, while RR produced the lowest R^2^ and the largest RMSE and MAE values, indicating its limited ability to capture the nonlinear relationships between the input variables and UCS. Overall, NRBO-XGBoost provided the most favorable balance between fitting accuracy, prediction error, and generalization performance.

### 3.3. SHAP analysis

**Fig 14:**
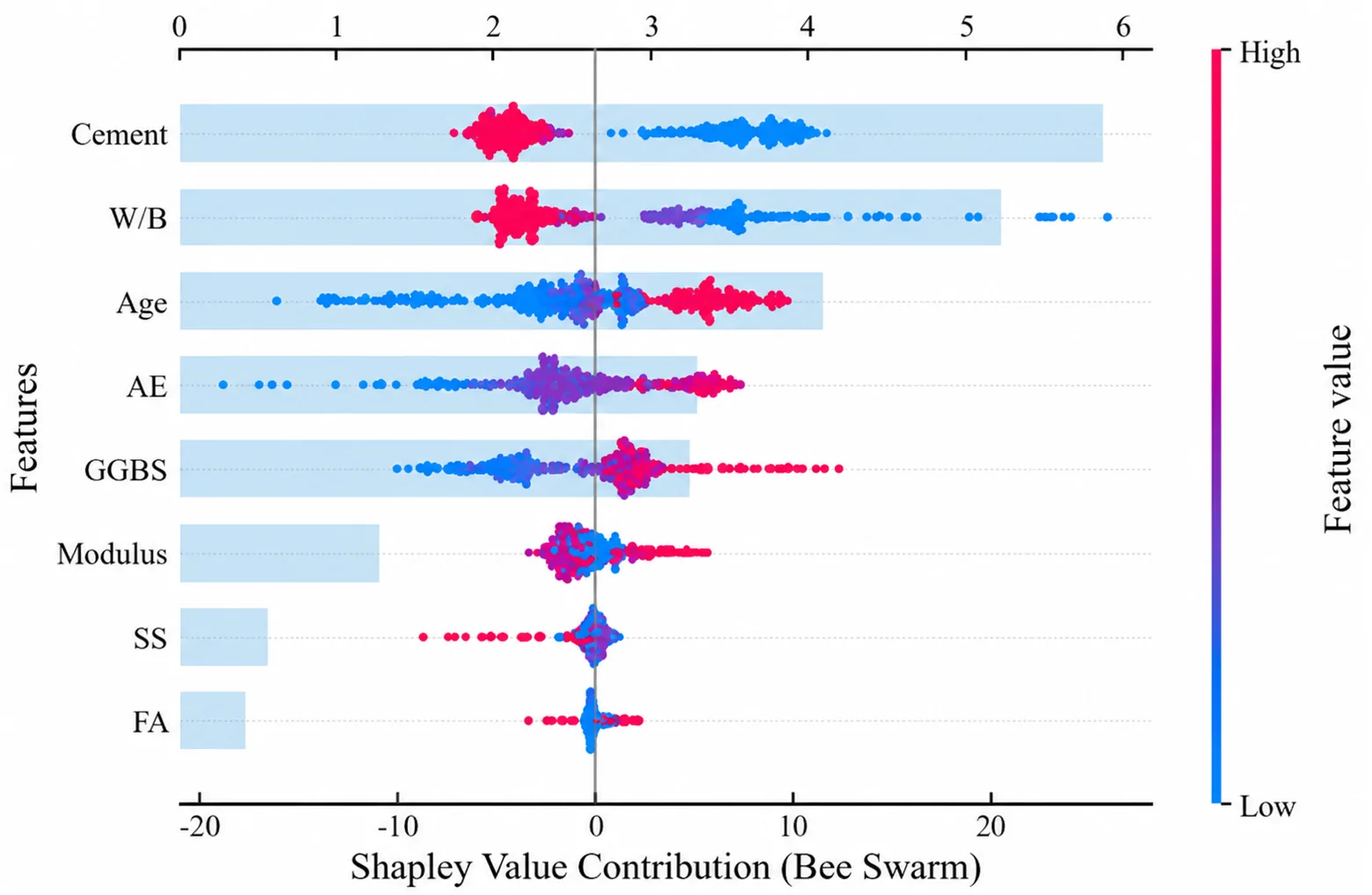
Shapley Value Contribution and Feature Importance Bee Swarm Plot for Features.

Based on the optimal NRBO-XGBoost model, SHAP analysis was performed to quantify the contribution of each input variable to the predicted UCS, as shown in Fig 14. The horizontal bars represent the mean absolute SHAP values and therefore indicate global feature importance, whereas the beeswarm distribution illustrates the direction and magnitude of each feature’s contribution. Cement content was identified as the most influential variable, followed by W/B, curing age, AE, and GGBS. In contrast, Modulus, SS, and FA exhibited comparatively limited effects on the model output.

The SHAP value distributions further reveal distinct directional relationships. High cement contents and high W/B values were predominantly associated with negative SHAP values, indicating reductions in the predicted UCS. Conversely, greater curing age, AE, and GGBS contents generally produced positive SHAP contributions and therefore promoted strength development. Modulus exhibited a relatively weak and nonlinear influence, while SS and FA contributed only marginally across most samples. Overall, the results demonstrate that the predicted UCS is governed primarily by binder composition, water content, alkaline activation conditions, and curing age.

**Fig 15:**
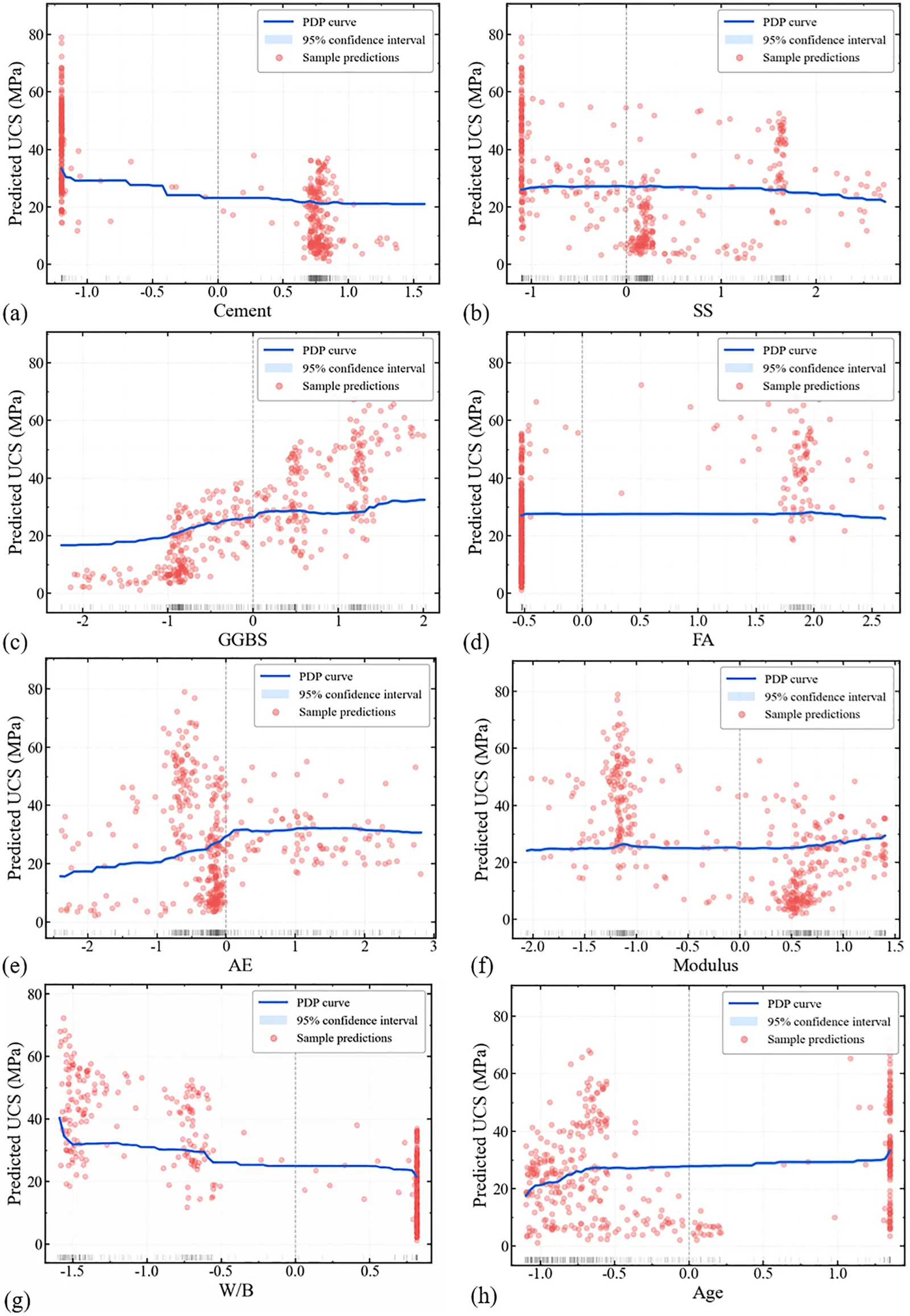
Partial dependence plots of input features for UCS prediction. (a) Cement. (b) SS. (c) GGBS. (d) FA. (e) AE. (f) Modulus. (g) W/B. (h) Age.

To further examine the marginal effects of the input variables on UCS prediction, partial dependence plots (PDP) were generated for the optimal NRBO-XGBoost model, as shown in Fig 15. The blue curves represent the average partial dependence, the shaded regions denote the 95% confidence intervals, and the rug marks indicate the distribution of feature values.

Cement and W/B exhibited predominantly negative effects on the predicted UCS. The predicted strength decreased rapidly at the lower end of the cement range and then declined gradually as cement content increased. Similarly, increasing W/B generally reduced the predicted UCS, confirming the adverse effect of excessive water content on strength development. In contrast, GGBS, AE, and curing age showed positive marginal effects. The predicted UCS increased markedly with GGBS and AE before approaching a plateau, indicating diminishing strength gains at higher levels. Curing age also produced a gradual increase in predicted UCS, consistent with the continued development of alkali-activation reactions over time. SS and FA displayed relatively flat partial dependence curves, suggesting limited individual contributions within the investigated ranges. Modulus exhibited a weak nonlinear effect, with only minor variation in predicted UCS across most of its range. Overall, the PDP results are consistent with the SHAP analysis, demonstrating that UCS prediction is primarily controlled by cement content, W/B, GGBS, AE, and curing age. However, variations near the sparsely sampled boundaries should be interpreted cautiously because predictions in these regions are supported by fewer observations.

## 4. Conclusions

This study addresses the challenges of predicting the compressive strength (UCS) of alkali-activated multi-waste grouting materials, which are influenced by multiple coupled factors and constrained by limited experimental data. A predictive framework integrating WGAN-GP data augmentation, NRBO hyperparameter optimization, and XGBoost was developed, with SHAP employed for systematic interpretability of the model’s decision process. Based on the findings, the main conclusions are as follows:

(1) WGAN-GP effectively captured the statistical characteristics of the original experimental dataset. The generated samples reproduced the principal marginal distributions, variable ranges, and inter-variable correlations of the experimental data. The mean difference between the correlation matrices of the original and generated datasets was only 0.0349, and no out-of-range input values or invalid UCS values were observed. These results confirm the statistical consistency and basic physical plausibility of the generated samples.
(2) WGAN-GP-based data augmentation improved the prediction accuracy and stability of the baseline XGBoost model. After introducing the generated samples, the testing R^2^ increased from 0.882 to 0.911, while RMSE and MAE decreased from 6.482 and 4.771 MPa to 5.615 and 3.627 MPa, respectively. The augmented dataset also produced more stable cross-validation results, indicating that data augmentation enriched the available feature space and reduced the adverse effects of the small experimental dataset.
(3) NRBO optimization further enhanced the predictive performance of XGBoost. The NRBO-XGBoost model trained on the augmented dataset achieved the best testing performance, with an R^2^ of 0.964, an RMSE of 3.411 MPa, and an MAE of 2.474 MPa, outperforming the baseline XGBoost, SVR, RF, LightGBM, and ridge regression models. On the completely independent validation set, the model maintained an R^2^ of 0.961, an RMSE of 2.84 MPa, and an MAE of 2.20 MPa, demonstrating satisfactory generalization capability and limited susceptibility to overfitting.
(4) SHAP and partial dependence analyses identified cement content, water-to-binder ratio (W/B), curing age, alkali equivalent, and GGBS content as the dominant variables governing UCS prediction. Higher W/B generally reduced the predicted UCS, whereas increasing curing age, alkali equivalent, and GGBS content promoted strength development within the investigated ranges. The apparent negative contribution of cement content should be interpreted together with its strong correlations with GGBS, activator modulus, and water content rather than as an isolated material effect.

Overall, the proposed framework demonstrates the complementary roles of generative data augmentation and intelligent hyperparameter optimization in improving strength prediction under small-sample conditions. It provides a reliable data-driven tool for preliminary mixture screening and proportion optimization of alkali-activated multi-source solid-waste grouting materials. Nevertheless, the applicability of the model remains limited to the compositional ranges and experimental conditions covered by the current database. Future studies should incorporate larger, multi-source, and multi-laboratory datasets, further examine the physical reliability of generated samples through compositional constraints, and introduce more comprehensive uncertainty and statistical analyses to improve the robustness and transferability of the proposed framework.

## Supporting information

S1 Data Original experimental dataset used in this study. The dataset contains 179 experimental records of mixture compositions, curing ages, and corresponding unconfined compressive strength values for alkali-activated multi-source solid-waste grouting materials.
(XLSX)
